# Medical and Physical Intelligence

**Published:** 1821-07

**Authors:** 


### Medical and Physical Intelligence.

---

*Continuation of the Analysis of the first Number of the Journal de Physiologie Experimentale.*

"*Experiments on Veratrine, or the active Principle of White Hellebore, Colchicum Autumnale, and Sabadilla.* By Mr. ANDRAL, jun."

WE shall adduce the principal facts stated in this memoir, without making any comments on them on this occasion, in conformity with our conduct in the analysis of the rest of the articles in this Journal.

It is stated, in some preliminary remarks, that the active principles of the three plants above enumerated,—which may be obtained in an alkaline form, like *picrotoxine* from the *cocculus indicus, strychine* from *nux vomica,* &c.—are *analogous** in the whole, and like the analogous active principles of nux vomica, cocculus indicus, &c. is of an alkaline nature.

The account of the experiments here under consideration is derived

---

* This word is commonly used by the French to signify *like* or *similar,* as well as in the sense of the term *analogous* as it is applied by English writers.

from Dr. MAGENDIE's Lectures on the Actions of Medicines. It is not, however, necessary for us to give an account of the experiments in detail: the author's general inferences from them will show the interesting facts they present. "It results from these experiments," the author says, "that veratrine exerts on the animal economy an action similar to that of the vegetables whence it is extracted: applied immediately to the tissues, it excites inflammation of them; injected into veins, it exerts an irritative agency on the large intestines. When the quantity of veratrine introduced into the alimentary canal is very small, it produces only local effects: when in a large quantity, it is absorbed, and produces tetanus; which, for stronger reasons, it also produces when it is directly injected into the veins.

*Note respecting the Nerves which go to the Mustachios of the Phoca*, by Mr. Andral, jun.—"Beneath the skin, on each side the muzzle, we see in the phoca, (as Mr. Andral says,) a very large nerve, which, having arrived at the origin of its enormous mustachios, is divided into as many filaments as there are bristles, nearly like the superior branch of the facial nerve is seen divided in man. This nerve evidently terminates in the mustachio of its side, to which it appears to be specially destined. Each of the filaments which result from its division, penetrates the *piliferous* capsule by its extremity, the most remote from the skin, (par son extremité la plus éloignée de la peau,)* is distributed in its interior, where it becomes impossible to follow it. The piliferous capsule is itself very strongly marked."

---

ON Saturday, the 19th of May, was held at the Freemason's Tavern, the fifth anniversary festival of the Medical Benevolent Society, under the patronage of his Royal Highness the Duke of Sussex.

His Royal Highness being unable, through indisposition, to honour the Society by his personal attendance, the chair was taken by the president, Dr. Latham, and supported by Sir John Doyle, bart. K.C. an honorary member, and Dr. Baillie. The company was numerous and highly respectable.

Though no collection was made, yet many very handsome donations, amounting to between 50*l.* and 60*l.* were announced by the treasurer; among which were ten guineas from Dr. Baillie, and the like sum from Sir Matthew Tierney, bart. M.D. The names of several gentlemen were declared as candidates to become members. It is truly gratifying to observe the flourishing state of this Society, which, though of only five years' duration, consists of more than two hundred members, and is daily increasing. There can be no doubt but that the benevolent purposes for which it was designed will be fully answered, and that the poorer and more afflicted members of the medical profession may look up to it with confidence, as offering them suitable means of relief under such circumstances of distress, as will too often occur, notwithstanding the most strenuous endeavours on their part to prevent them.

---

* We give the original, lest we should be taxed with making the account obscure.—EDIT.

*On the frauds practised by persons to gain admission to the Medical Profession, and the necessity of counteracting them; by an* Old Practitioner.

The criminal indifference of the members of the medical profession to the introduction of new members by fraudulent means, has been a source of much public mischief, and has degraded and injured the profession; if some check is not put to this practice, what confidence can the world have in medical science, or, how are legally and duly qualified members of the profession to be distinguished and rewarded for their skill and acquirement? I live in the neighbourhood of a practitioner who has taken upon himself to qualify pupils for medical practice, in the short space of a few months. As the law precludes all persons from admission into the chartered bodies of surgeons and apothecaries, but such as have *(bona fide)* served a regular term of five years as apprentices, I feel myself bound to prevent, as far as is in my power, the admission of all candidates, who are endeavouring, by surreptitious means, to gain such admission into the profession.

If any member of the medical profession (tired of his own pursuits,) should seek admission to the law or the church, he would find the members of these professions too much alive to their own respectability and interest, to admit him without his having first regularly submitted to the probatory studies, and spent the ordinary time prescribed by law, in their pursuits. The science of medicine is not less important in the scale of usefulness than the former; indeed, the man of rank and the poorest cottager, are alike interested in the education of medical practitioners, for no man knows how soon he may be placed under the care of one of that body; therefore every one is interested in their being all duly qualified. There will be diversity of talent in the members of the profession; but no person should, from false notions of liberality, be allowed to practise, whose mind has not been stored by long and patient observation. No parrot-taught pupils, no persons who have not legitimate claims should be eligible for practice. I suspect, that in some cases I have to present, the fraud of antedating the indenture has been used. I beg to be informed, through your Journal, who are the proper authorities to prevent the admission of such persons, as it is my intention to lodge such information, in every case that comes under my observation, as will prevent surreptitious introduction to the profession; and I trust that my example will be followed by all the members of our fraternity.

*Somersetshire, June* 11, 1821.

*** We did not receive this paper until it was too late to insert it in the department for original communications; and, being willing to favour, without delay, the acquisition of the information the writer requires, we give it in this place. Our Journal is open for the reception of any reply to it; but we believe the following is the state of the laws relative to the subject:—that there is no law to prevent any person practising medicine in England, excepting as an *apothecary*. The jurisdiction of the London College of Physicians, relates only to a certain extent round London, and to the practice of any one as a *physician*. Against any person exercising surgery there is no law at all vested in any body or college. The Company of Apothecaries has the power to prevent any person practising as an apothecary in England and Wales; and it is the officers of this company, (the master and wardens, we suppose,) that alone are qualified to prosecute delinquents. Information of infringements of their charter may, therefore, be sent to those officers; but if they do not choose to undertake the prosecution, there is no means of remedying, in the evil complained of by our correspondent, this consequence of British liberty.—EDITOR.

*On vertiginous Indigestion in the Horse;* (extracted from a Report made by Professors BURDIN and DUPUY, on some observations by Mr. MANGIN.)

IN the course of the year 1817, the abdominal vertigo, commonly termed simply *vertigo*, (*staggers*,) made great ravages amongst horses in the departments of the Moselle and the Meuse. Mr. Mangin, having had occasion to treat a great number of these animals, was soon led to recognize the insufficiency of the remedies commonly employed. He was called to a horse affected with well characterized vertiginous indigestion; this animal had neither eaten nor drank for twelve hours. He gave him two drachms of tartar emetic, with four ounces of sulphate of magnesia, in a pint of warm water. This potion was repeated thrice in the space of fifteen hours. He placed a seton in each thigh, and had the limbs rubbed with oil of turpentine. The animal died on the ensuing day. He treated a second and a third horse in the same way, with similar results. A neighbouring veterinary surgeon, who employed similar means, had no better success, having had four horses die under his care, in six-and-thirty hours. Mr. Mangin having remarked, on opening several of the animals after death, that the stomach was empty, whilst the large intestines were filled with a very large quantity of fecal matters, suspected that these intestines might be the seat of the disease; and, considering that tartar emetic acts especially on the stomach, he determined to employ purgatives in large doses. Soon after he had made this resolution, a horse of six years old, affected with the disease, was brought to him. He administered, in the space of twelve hours, four potions, each composed of two ounces of aloes, and three ounces of sulphate of magnesia. Fifteen hours after the first dose, the horse voided a large quantity of feces. He then gave him decoctions of linseed, with two drachms of camphor, from time to time. The animal suffered great weakness, which, however, gradually disappeared, and on the sixth day afterwards he was considered as perfectly well. Three other horses were afterwards cured by the same means; and after this a farmer, who had lost four under the use of bleeding, antimony, and setons, in this affection, consulted Mr. Mangin for two others, which were cured by purgatives.

Mr. Mangin attributes the disease to the hay made in the year 1816, in a very rainy season; and in some other cases, in the next year, to the use of green trefoil, as the sole food. Some other horses affected with it had eaten copiously of new oats.

The Reporters blame Mr. Mangin for having administered so large a quantity of aloes in so short a period; but Mr. Mangin apologizes for his practice, (which would excite excessive purging in a healthy horse,) by observing, that the horse is here in a comatose state; in consequence of which, purgative medicines do not act with their ordinary energy.

The Reporters think that they can account for the advantages derived from Mr. Mangin's treatment, by some considerations drawn from the anatomy of the horse. The stomach of this animal is of very small capacity; it will contain, at most, but twelve pints of water, in a horse of the middling size; besides this, the left half of the stomach is lined by a white, thick, and callous membrane, similar to that also lining the œsophagus; and this half is separated from the right portion by a fringed sort of valvular process; the right portion is lined with a very vascular mucous membrane, and it is in this alone that digestion is operated. There is, without doubt, some difficulty in reconciling this small capacity with the large quantity of food which this animal will take at an ordinary meal. It seems necessary to suppose that the solid food is very promptly converted into chyme, and that the drink is absorbed with great rapidity.

If the stomach be very small, the intestines, on the contrary, are very voluminous. It is in the latter that the solid parts of the food are accumulated, and where they sojourn for a greater or less length of time. This fact would incline us to the use of purgatives, even though experience had not proved that they disembarrass the stomach as well as the intestines; and, as it is well known, tartar emetic does not produce vomiting in the horse.

Professor Dupuy remarks, that many different diseases are ordinarily confounded under the name of vertigo: in some horses who had been considered to die from this affection, he has found the internal membrane of the heart, the aorta, and the venæ cavæ, thickened and very much injected with blood; and this coloration of the membrane has not disappeared, although the parts have been macerated for four or five days in water, frequently renewed. The same membrane will appear of a violet hue, in many other diseases; but here, (he says,) the colour depends on "a simple precipitation of the colouring matter of the blood, which is deposited on the membrane." Professor Dupuy intends to treat more at length on these subjects in a memoir which he will shortly read to the Medical Society.

---

A HORSE, after a long abstinence, during some moderate work, ate, with a sort of voracity, a large quantity of herbage, composed, in part, of the plant *sinapis arvensis,* and afterwards voided, in the space of twelve hours, nearly six pailfuls of saliva. Three cows, who also ate of this plant, manifested similar effects, though to less extent. Two asses, for the sake of the experiment, were forced, by privation of other food, to eat of this vegetable; but they presented no such effects: the quantity of it eaten by them was, however, but very small.

(*Compte rendu des travaux de l'Ecole royale d'economie rurale et veterinaire de Lyon, en* 1820.)

---

*Notice of a prolific cross-breed between the common Cat and the Pine-Martin, (Mustela Martes.)*—We find by the *Bibliotheque Universelle,* that there has been lately presented to the Imperial Society of Natural History, of Moscow, an animal which appears to be a cross-breed, formed by the meeting of the common cat and the pine-martin, and the fur of which promises to be a valuable article of commerce. The specimen presented to the Society was sent from the government of Penza, where the pine-martin is very abundant. The following history is given of the cross-breed.—A domestic cat disappeared from a house in Penza, and returned in some days in a state of impregnation. At the usual period the cat littered four young ones, two of which very much resembled the martin. Their claws were not retractile, as in the cat, and the snout was elongated like that of the martin. The two others, of the same litter, more nearly resembled the cat, as they had retractile claws and a round head. All of them had the black feet, tail, and ears of the martin; and they killed birds and small animals, more for the pleasure of destroying them than for food. The proprietor endeavoured to multiply this bastard race, and to prevent their intermixing with the other domestic cats; and his endeavours were completely successful. In the space of a few years he reared more than a hundred of these animals, and he made a very beautiful article of furriery of their skins. The specimen presented to the Society was of the third or fourth generation, and it retained all the characters of the first. The fur is as beautiful and as silky as that of the pine-martin, and it may, with some care, become an interesting object for commerce.

---

*Description of the Mummy-Pits at Thebes, by* Mr. Belzoni.—"The passage where the bodies are is roughly cut in the rocks, and the falling of the sand from the ceiling of the passage, causes it to be nearly filled up. In some places there is not more than a foot left, which you must pass through, creeping like a snail on pointed stones that cut like glass. After getting through these passages, some of them 200 or 300 yards long, you generally find a more commodious place, perhaps high enough to sit. But what a place of rest! surrounded by bodies, by heaps of mummies in all directions, which impressed me with horror. The blackness of the wall, the faint light given by the candles and torches for want of air, the different objects that surrounded me seeming to converse with each other; and the Arabs, with the candles or torches in their hands, naked, and covered with dust, themselves resembling living mummies, formed a scene that cannot be described. After the exertion of entering into such a place, through a passage of 80, 100, 300, or perhaps 600 yards, nearly overcome, I sought a resting-place, I found one, and contrived to sit; but when my weight bore on the body of an Egyptian, it crushed it like a band-box. I instantly had recourse to my hands to sustain my weight, but they found no better support, so that I sunk altogether among the broken mummies with a crash of bones, rags, and wooden cases, which raised such a dust as kept me motionless for a quarter of an hour, waiting till it subsided again. I could not remove from the place, however, without increasing it, and every step I took I crushed a mummy in some part or other. Once I was conducted through a passage no wider than that of the body, and choked with mummies, and I could not pass without putting my face in contact with some decaying Egyptian; but as the passage inclined downwards, my own weight helped me on; however, I could not avoid being covered with bones, legs, arms, and heads rolling from above. Thus, I proceeded from one cave to another, all full of mummies, piled up in various ways, some standing, some lying, and some on their heads." *Narrative of Operations and Discoveries in Egypt*, p. 156.

---

### *Bills of Mortality, &c. for London, of the Year* 1820.

| DISEASES. | |
|---|---|
| Abscess | 90 |
| Apoplexy | 233 |
| Asthma | 702 |
| Bedridden | 1 |
| Cancer | 69 |
| Childbed | 208 |
| Consumption | 3959 |
| Convulsions | 3066 |
| Croup | 104 |
| Diabetes | 1 |
| Diarrhœa | 9 |
| Dropsy | 791 |
| Dropsy in the Brain | 332 |
| Dropsy in the Chest | 90 |
| Dysentery | 6 |
| Epilepsy | 9 |
| Eruptive Diseases | 12 |
| Erysipelas, or St. Anthony's Fire | 13 |
| Fever | 1109 |
| Fever (Typhous) | 47 |
| Fistula | 3 |
| Flux | 6 |
| Gout | 48 |
| Hæmorrhage | 25 |
| Whooping Cough | 794 |
| Inflammation | 1247 |
| Inflammation of the Liver | 66 |
| Insanity | 223 |
| Jaundice | 77 |
| Jaw Locked | 1 |
| Measles | 720 |
| Miscarriage | 3 |
| Mortification | 220 |
| Old Age and Debility | 2220 |
| Palsy | 176 |
| Rheumatism | 10 |
| Rupture | 32 |
| Scrofula | 7 |
| Small-pox | 792 |
| Sore Throat and Quinsey | 15 |
| Spasm | 46 |
| Still-born | 725 |
| Stone | 18 |
| Stoppage in the Stomach | 8 |
| Suddenly | 248 |
| Teething | 409 |
| Thrush | 79 |
| Venereal | 11 |
| Worms | 18 |
| Total of Diseases, | 19098 |

| CASUALTIES. | |
|---|---|
| Burnt | 22 |
| Choked | 1 |
| Drowned | 96 |
| Excessive Drinking | 2 |
| Executed* | 10 |
| Found Dead | 5 |
| Fractured | 2 |
| Frightened | 1 |
| Frozen | 1 |
| Killed by Falls and several other Accidents | 78 |
| Murdered | 1 |
| Scalded | 1 |
| Strangled | 2 |
| Suffocated | 7 |
| Suicides | 21 |
| Total of Casualties, | 250 |

| | | | |
|---|---|---|---|
| Christened | Males | 11,993 | In all, 23,158 |
| | Females | 11,165 | |
| Buried | Males | 9,794 | In all, 19,348 |
| | Females | 9,554 | |

Whereof have died:

| | | | |
|---|---|---|---|
| Under Two Years of Age | 4758 | Sixty and Seventy | 1632 |
| Between Two and Five | 1975 | Seventy and Eighty | 1208 |
| Five and Ten | 887 | Eighty and Ninety | 662 |
| Ten and Twenty | 667 | Ninety and a Hundred | 119 |
| Twenty and Thirty | 1484 | A Hundred | 2 |
| Thirty and Forty | 2006 | A Hundred and One | |
| Forty and Fifty | 2069 | A Hundred and Two | 1 |
| Fifty and Sixty | 1878 | A Hundred and Three | |

Increased in the Burials this year, 120.

* There have been executed in London and the county of Surrey, 38; of which number, 10 only have been reported to be buried within the Bills of Mortality.

---

### MONTHLY CATALOGUE OF MEDICAL BOOKS.

### METEOROLOGICAL JOURNAL.

By Messrs. WILLIAM HARRIS and Co. 50, Holborn, London.

*From May* 20 *to June* 19, *inclusive.*

| Day of Month. | Moon. | Rain gauge | THERM. | | | BAROM. | | DeLuc's Hygro. | | WIND. | | ATMOSPHERIC VARIATION. | | |
|---|---|---|---|---|---|---|---|---|---|---|---|---|---|---|
| May | | | | | | | | | | | | | | |
| 20 | | | 48 | 52 | 41 | 30·20 | 30·21 | 57 | 57 | NE | NE | Cloudy | | |
| 21 | | ·08 | 45 | 53 | 40 | 30·00 | 30·05 | 60 | 57 | E | ENE | Fine | Cloud. | Fine |
| 22 | | | 43 | 54 | 43 | 30·02 | 29·93 | 55 | 53 | ENE | NE | Fine | | |
| 23 | | ·03 | 47 | 55 | 38 | 29·78 | 29·91 | 54 | 55 | NE | N | Cloudy | Sho'ry | |
| 24 | ☽ | ·03 | 44 | 52 | 42 | 29·96 | 30·04 | 55 | 53 | N | N | Fine | Sho'ry | Cloud. |
| 25 | | ·07 | 49 | 55 | 40 | 29·96 | 29·76 | 54 | 55 | WSW | W | Fine | | Rain |
| 26 | | ·02 | 42 | 58 | 39 | 29·78 | 29·86 | 54 | 54 | NW | NNW | Fine | Sh. Sleet | Cloud. |
| 27 | | ·10 | 48 | 58 | 43 | 29·92 | 29·95 | 53 | 55 | NW | SW | Fine | Sho'ry | Rain |
| 28 | | ·09 | 49 | 58 | 44 | 29·95 | 30·07 | 55 | 55 | WNW | WSW | Fine | Sho'ry | Cloud. |
| 29 | | | 48 | 57 | 48 | 30·12 | 30·22 | 56 | 53 | W | NE | Cloudy | Fine | Cloud. |
| 30 | | | 54 | 58 | 45 | 30·23 | 30·21 | 53 | 54 | E | ENE | Fine | | |
| 31 | ● | | 51 | 57 | 50 | 30·15 | 30·11 | 54 | 55 | NE | NE | Fine | | |
| Jun. | | | | | | | | | | | | | | |
| 1 | | | 54 | 63 | 54 | 30·06 | 30·06 | 58 | 57 | E | ENE | Fine | | |
| 2 | | | 62 | 68 | 54 | 30·02 | 29·96 | 55 | 51 | NE | NE | Fine | | |
| 3 | | | 59 | 66 | 58 | 29·90 | 29·81 | 55 | 56 | ESE | ENE | Cloudy | Fine | |
| 4 | | ·21 | 56 | 62 | 54 | 29·71 | 29·70 | 59 | 56 | ENE | WNW | Rain | Fine | |
| 5 | | ·30 | 58 | 68 | 55 | 29·76 | 29·82 | 57 | 55 | WNW | SW | Fine | | Rain |
| 6 | | ·24 | 58 | 69 | 56 | 29·88 | 29·85 | 57 | 58 | W | SW | Cloudy | Rain | Sho'ry |
| 7 | ☾ | ·41 | 59 | 62 | 53 | 29·82 | 29·63 | 57 | 57 | SW | WSW | Cloudy | Rain | |
| 8 | | ·25 | 54 | 54 | 45 | 29·63 | 29·85 | 58 | 56 | NNE | NNE | Rain | Sho'ry | Cloud. |
| 9 | | | 52 | 56 | 46 | 29·85 | 29·81 | 54 | 55 | W | W | Cloudy | Rain | Sho'ry |
| 10 | | ·24 | 50 | 52 | 44 | 29·76 | 29·84 | 55 | 56 | W | NNE | Cloudy | Sho'ry | Rain |
| 11 | | ·29 | 49 | 51 | 46 | 29·90 | 30·10 | 56 | 57 | NE | N | Fine | Sho'ry | Rain |
| 12 | | | 51 | 57 | 48 | 30·20 | 30·28 | 56 | 56 | NE | NNE | Fine | Cloud. | |
| 13 | | | 54 | 58 | 46 | 30·25 | 30·27 | 56 | 56 | N | NNE | Cloudy | | Fine |
| 14 | | | 52 | 59 | 50 | 30·30 | 30·36 | 55 | 56 | NNE | NNE | Cloudy | Fine | |
| 15 | ○ | | 54 | 63 | 54 | 30·34 | 30·27 | 57 | 56 | NNE | NE | Fine | | |
| 16 | | ·05 | 56 | 60 | 50 | 30·23 | 30·28 | 57 | 56 | NE | NNE | Misty Ra | Fine | Cloud. |
| 17 | | | 54 | 59 | 51 | 30·31 | 30·32 | 56 | 56 | ENE | ENE | Cloudy | Fine | Cloud. |
| 18 | | | 53 | 63 | 49 | 30·33 | 30·31 | 56 | 57 | NE | NNE | Cloudy | Fine | |
| 19 | | ·02 | 53 | 64 | 50 | 30·25 | 30·16 | 56 | 53 | NE | NNE | Fine | | |

The quantity of rain fallen in the month of May, is 1 inch and 59-100ths.

